# Validity and Reliability of Pre-matriculation and Institutional Assessments in Predicting USMLE STEP 1 Success: Lessons From a Traditional 2 x 2 Curricular Model

**DOI:** 10.3389/fmed.2021.798876

**Published:** 2022-01-27

**Authors:** Nitin Puri, Michael McCarthy, Bobby Miller

**Affiliations:** Office of Medical Education, Joan C. Edwards School of Medicine, Marshall University, Huntington, WV, United States

**Keywords:** predict accuracy, USMLE STEP 1, institutional assessments, success, curricular model

## Abstract

**Purpose:**

We have observed that students' performance in our pre-clerkship curriculum does not align well with their United States Medical Licensing Examination (USMLE) STEP1 scores. Students at-risk of failing or underperforming on STEP1 have often excelled on our institutional assessments. We sought to test the validity and reliability of our course assessments in predicting STEP1 scores, and in the process, generate and validate a more accurate prediction model for STEP1 performance.

**Methods:**

Student pre-matriculation and course assessment data of the Class of 2020 (*n* = 76) is used to generate a stepwise STEP1 prediction model, which is tested with the students of the Class of 2021 (*n* = 71). Predictions are developed at the time of matriculation and subsequently at the end of each course in the programing language R. For the Class of 2021, the predicted STEP1 score is correlated with their actual STEP1 scores, and data agreement is tested with means-difference plots. A similar model is generated and tested for the Class of 2022.

**Results:**

STEP1 predictions based on pre-matriculation data are unreliable and fail to identify at-risk students (R2 = 0.02). STEP1 predictions for most year one courses (anatomy, biochemistry, physiology) correlate poorly with students' actual STEP1 scores (R^2^ = 0.30). STEP1 predictions improve for year two courses (microbiology, pathology, and pharmacology). But integrated courses with customized NBMEs provide more reliable predictions (R^2^ = 0.66). Predictions based on these integrated courses are reproducible for the Class of 2022.

**Conclusion:**

MCAT and undergraduate GPA are poor predictors of student's STEP1 scores. Partially integrated courses with biweekly assessments do not promote problem-solving skills and leave students' at-risk of failing STEP1. Only courses with integrated and comprehensive assessments are reliable indicators of students' STEP1 preparation.

## Introduction

The United States Medical Licensing Examination (USMLE) STEP1 is the first of the four licensure examinations a medical graduate must pass to become a practicing allopathic medical doctor in the United States. This highly integrated exam assesses clinical problem-solving and the student's ability to apply basic science concepts to principles and mechanisms associated with the organ systems' structure, function, disease, and therapeutics. USMLE STEP1 scores are also used as a promotion requirement from the pre-clerkship to the clerkship curriculum; and as a screening tool for competitive residency placements (1, 2).

Although USMLE has recently changed the STEP1 score-report format from a three-digit score to a pass/fail report, it remains a crucial milestone for the academic progression of a medical student. The student's STEP1 status, and the number of attempts involved in passing the exam, are likely to have a continued impact on the residency selection process. Considering the preceding, success on this standardized examination is of paramount importance to medical students and the medical institution, with significant individual and institutional effort spent on preparing for this examination.

The information to prepare for the USMLE STEP1 is primarily acquired during the first 2 years of the pre-clerkship phase of the undergraduate medical curriculum. Joan C. Edwards School of Medicine (JCESOM) at the Marshall University utilizes the traditional 2 x 2 curricular model to instruct medical students. The MS1 (year 1) curriculum focuses on anatomy, physiology, and biochemistry pedagogy. The MS2 (year 2) curriculum is centered on pedagogy in microbiology, pathology of disease, and modes of therapy. Medical students' comprehension of this information is evaluated using institutional assessments created by the instructors and are primarily specific to each discipline. Students' performances on institutional assessments are included in prediction modeling, which could effectively chart individual students' academic progress, including STEP1 performance. Such predictions may help identify struggling students early in the pre-clerkship curriculum to intervene in a timely fashion.

However, as the USMLE STEP1 has evolved, JCESOM has encountered one challenge; reliably predicting a medical student's performance on the STEP1 examination, despite utilizing prediction modeling (3). For example, students who excel in the first 2 years of pre-clerkship education and are predicted to pass STEP1 fail or underperform, and vice versa. Consequently, medical students cannot reflect on their respective strengths and weaknesses to better prepare for their licensure examination. This also leaves the medical school ill-equipped to focus valuable resources or early interventions on students at true risk of failing STEP1. Based on the constructive alignment theory by Briggs (4), we hypothesized that medical school assessments focused on the recall of information, regardless of pedagogy, are not reliable indicators of student's STEP1 performance. On the other hand, integrated courses with an assessment focus on problem-solving, similarly aligned to national boards, may provide robust predictive analytics of students' future performance.

There is also a considerable national debate on the utility of MCAT and/or GPA in predicting student outcomes on medical school and licensure examinations. The data ranges from showing modest (5, 6) correlation between MCAT and STEP 1 to significant over predictions, based on the institution and ethnicity of the student (7, 8). Notably, most studies showing a positive correlation between MCAT and STEP1 use data from institutions with average MCAT in the mid to high-score ranges (>500). The average MCAT for incoming students at the JCESOM, Marshall University is between 501 and 502. Additionally, a recent multivariable study across multiple medical schools, using median reported data, found a significant correlation between MCAT and STEP1 (9). This could imply that MCAT scores may correlate with STEP1 performance for a cohort; higher average MCAT correlating with higher average STEP1 scores but may not be an effective tool to calculate risk for an individual student.

Hence, the focus of the study is to assess the validity and reliability of pre-matriculation and course-assessment data in predicting the future performance of our students at the USMLE STEP 1 licensure examination. We test the hypothesis by employing stepwise prediction modeling and correlating students' actual performance on the USMLE STEP 1 exam with their predicted scores.

## Materials and Methods

All student data in the study is de-identified before use. Joan C. Edwards School of Medicine's mission is to train physicians for the state of West Virginia. Our admissions process gives preference to W.V. residents and to students with significant ties to the state. Hence, our student cohort is largely from the state. Our student cohort is also relatively stable and evenly split between men and women. Since only a few students matriculate each year with advanced degrees, like post-baccalaureate masters, BCPM GPA is used in all prediction models.

USMLE STEP1 predictions for the Class of 2021 are based on the Class of 2020 (*n* = 76). STEP1 predictions for the Class of 2022 are based on data from the class of 2021 (*n* = 72). We use this “yearly” prediction modeling due to incremental changes in the M.D. curriculum each year, especially in the type and placement of assessments in the pre-clerkship curriculum.

Additionally, pre-matriculation data shifted in 2015 with the introduction of the new three-digit MCAT score. Class of 2020 is the first cohort with a three-digit MCAT for matriculating students. Pre-admission data is extracted from the American Medical College Application Service (AMCAS) database for students in the Class of 2020 who had subsequently taken United States Medical Licensing Examination (USMLE) Step 1 (*n* = 76). Only objective, academic data points are considered for generating prediction models.

This same cohort is followed through all MS1 and MS2 courses, and their course-exams results are tabulated along with their USMLE STEP1 scores. For generating STEP1 score-predictions for the Class of 2021, the data above for the Class of 2020 (training set) is subjected to linear regression modeling at each milestone with the USMLE STEP1 as the outcome. Models are computed in the programming language R (equations detailed in the supplemental section).

For the Class of 2021 (*n* = 72), the predicted STEP1 score (from the regression model above) generated at the end of each prediction point (course end) is plotted against the actual STEP1 score of the student. Means-difference plots (Bland Altman plots) show the agreement (10) between the predicted and actual STEP1 scores at each student's measured point.

For the Class of 2021, curricular modification included the inclusion of course-end customized NBMEs for the MS2 courses. Correlation and regression analysis of student performance on these NBMEs and STEP1 is also shown.

For the Class of 2022, the predicted STEP1 score (from the regression model above) generated at the end of each “prediction-point” is plotted against the actual STEP1 score of the student.

This study (IRB # 1630008-1) has been approved by the Marshall University Institutional Review Board under the exempt approval status.

## Results

### Pre-matriculation Data Does Not Accurately Predict STEP1 Score

**Figure 1:**
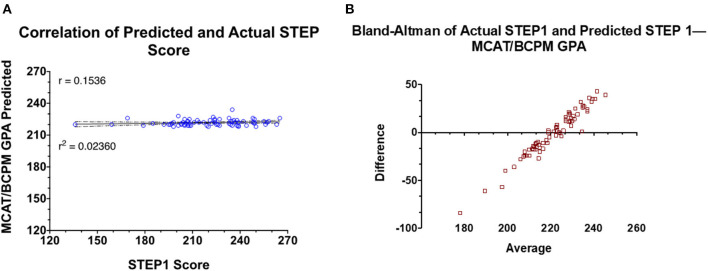
Prediction model generated based on retrospective data of the Class of 2020 is used to predict STEP1 scores for the Class of 2021. **(A)** Correlation and regression analysis of actual vs. predict STEP1 scores for the class of 2021 (*n* = 72). **(B)** Means-difference plot for the same cohort with predicted and actual STEP1 scores.

As shown in Figure 1, STEP1 score predictions generated by linear modeling three-digit MCAT scores and the undergraduate BCPM GPA of the candidates do not correlate well with the actual STEP1 scores of the candidates. The mean-difference plot highlights that MCAT/BCPM overpredict STEP1 scores for students scoring below the school mean (220) and underpredicts for students scoring above.

### STEP1 Predictions Based on MS1 Course Exams Have a Weak Correlation With STEP1

Similar to MCAT/BCPM GPA, STEP1 predictions are generated at the end of each MS1 course. The predicted STEP1 scores are correlated with actual STEP1 scores for the class of 2021—

**Figure 2:**
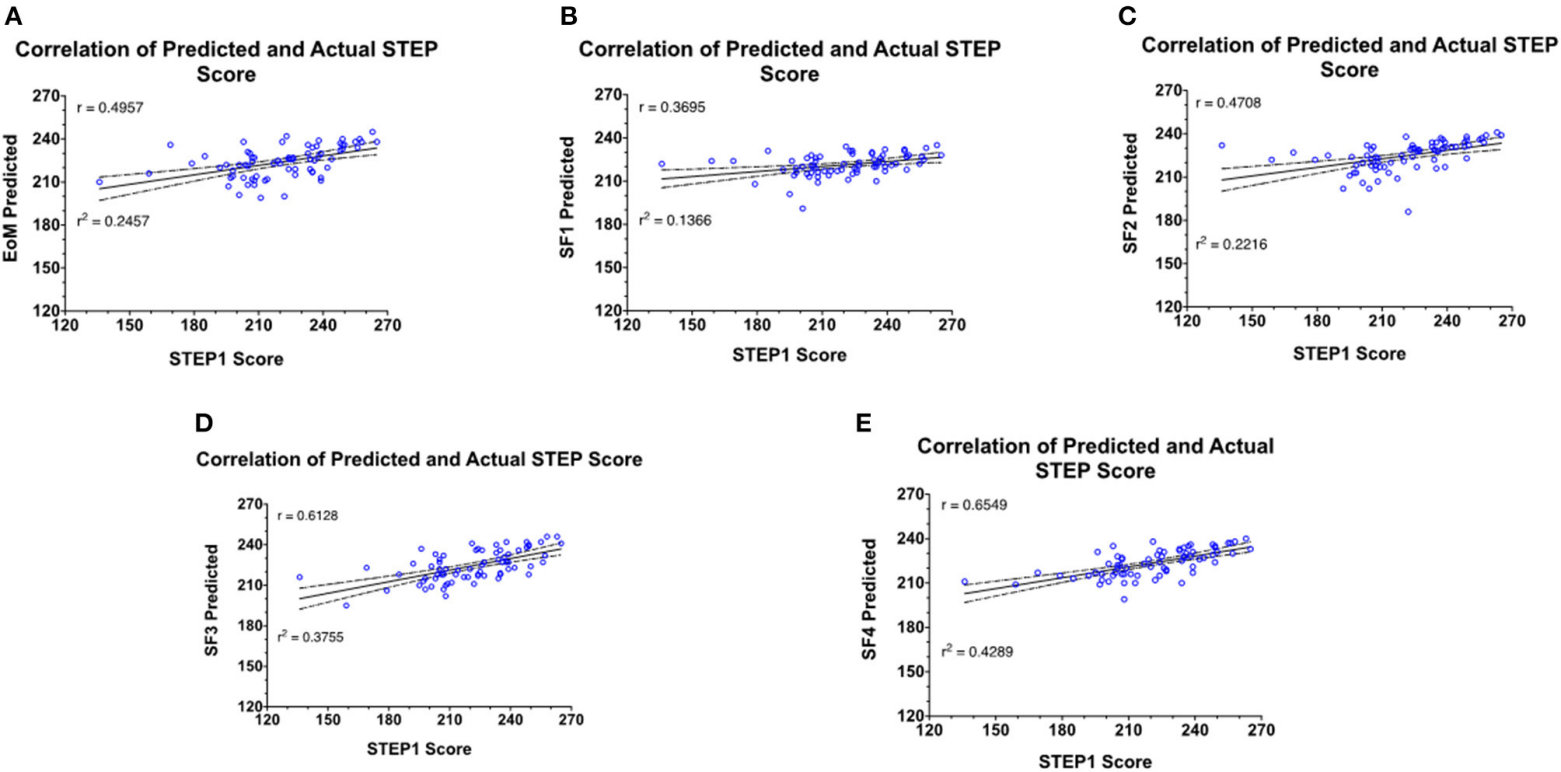
Prediction model generated based on retrospective data of the Class of 2020 is used to predict STEP1 scores for the Class of 2021. Predictions are generated at the end of every MS1 course. **(A–E)** Correlation and regression analysis of actual vs. predict STEP1 scores for the class of 2021 (*n* = 72) at each prediction point.

**Figure 3:**
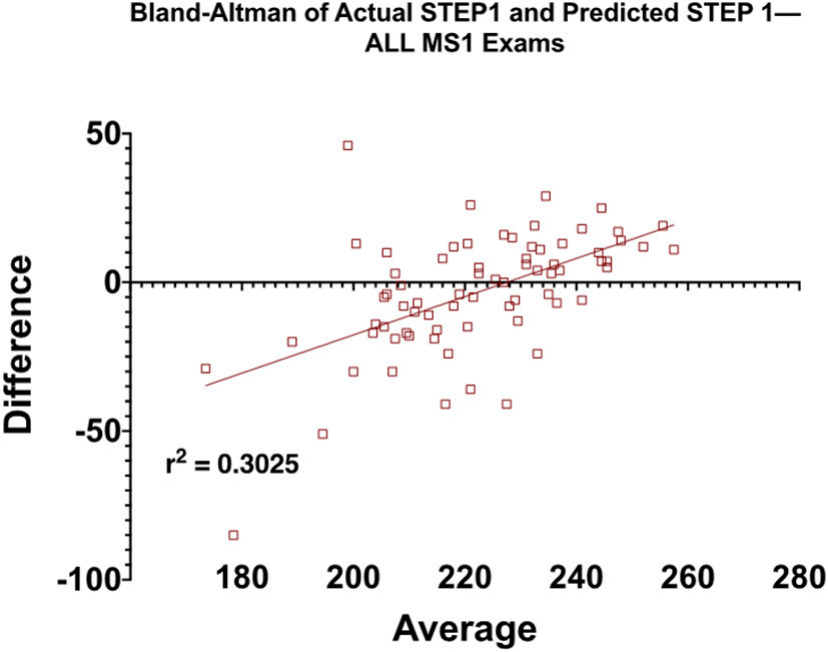
Means-difference analysis of predicted and actual STEP1 scores for the Class of 2021 (*n* = 72) at the end of MS1, pre-clerkship curriculum. Adjusted R^2^ for the regression analysis is also shown on the graph.

1. Elements of Medicine (EoM) course (Figure 2A): the first course of the M.D. curriculum is primarily focused on the pedagogy of biochemistry, cell biology, and genetics (R^2^ = 0.2457).
2. Structure and Function 1 (SF1) course (Figure 2B): the second course of the M.D. curriculum is primarily focused on the pedagogy of the musculoskeletal system and is the first course of the curriculum with anatomy labs (R^2^ = 0.1366). Notably, this correlation is weaker than at the end of the first course of the M.D. curriculum.
3. Structure and Function 2 (SF2) course (Figure 2C): the third course of the M.D. curriculum is primarily focused on the pedagogy of anatomy and physiology of the nervous system (R^2^ = 0.2216).
4. Structure and Function 3 (SF3) course (Figure 2D): the fourth course of the M.D. curriculum is primarily focused on the pedagogy of anatomy and physiology of cardiovascular, renal, and respiratory systems (R^2^ = 0.3755).
5. Structure and Function 4 (SF4) course (Figure 2E): the fifth and final course of the MS1, MD curriculum is primarily focused on the pedagogy of anatomy and physiology of gastrointestinal, genitourinary, and endocrine systems (*R*^2^ = 0.4289).
6. Figure 3 shows the predicted STEP1 score for all MS1 exams vs. actual STEP1 scores for the Class of 2021. Combined mean-difference and correlation plot highlights that at the end of the MS1 curriculum, STEP1 prediction models overpredict scores for most students scoring less than school average on STEP1 (220). MS1 exams combined underpredict scores for students performing better than the school average on USMLE STEP1.

### Regression and Means-Difference Plots of MS2 Course STEP1 Predictions With Actual STEP1 Scores

As before, STEP1 predictions are generated at the end of each MS2 course and correlated with actual STEP1 scores for the Class of 2021. Prediction modeling is based on the course exam and STEP1 scores of the Class of 2020. Means-difference (Bland Altman) plots are constructed at each prediction point—

**Figure 4:**
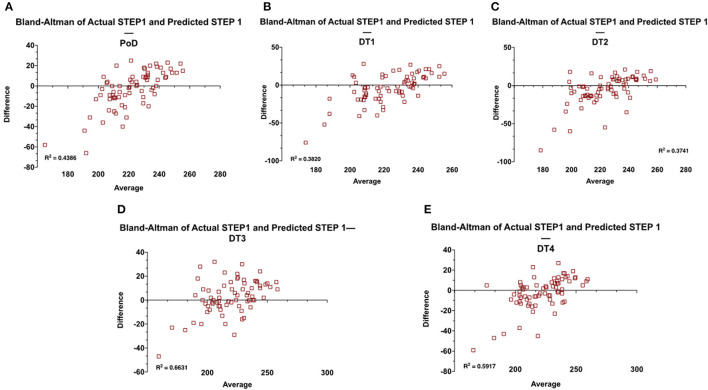
Means-difference analysis of predicted and actual STEP1 scores for the Class of 2021 (*n* = 72) at the end of each MS2 course of the pre-clerkship curriculum **(A–E)**. Adjusted R^2^ for the regression analysis is also shown on the graphs.

1. Principles of Disease (PoD) course (Supplementary Figure 1A, Figure 4A): the first course of the MS2 MD curriculum is primarily focused on the pedagogy of microbiology, immunology, and general pathology (*R*^2^ = 0.4386). The Means-difference plot shows over predictions for students scoring below the national mean on STEP1 (≈231).
2. Disease and Therapeutics 1 (DT1) course (Supplementary Figure 1B, Figure 4B): the second course of the MS2 MD curriculum is primarily focused on the pedagogy of the diseases of the musculoskeletal and the hematopoietic systems (*R*^2^ = 0.3820). As before, the means-difference plot shows over predictions for students scoring below the national mean on STEP1 (≈231).
3. Disease and Therapeutics 2 (DT2) course (Supplementary Figure 1C, Figure 4C): the third course of the MS2 MD curriculum is primarily focused on the pedagogy of the diseases of the nervous system and behavioral health (*R*^2^ = 0.3741). The Means-difference plot shows mostly overpredicted scores for all ranges of STEP1 scores.
4. Disease and Therapeutics 3 (DT3) course (Supplementary Figure 1D, Figure 4D): the penultimate course of the MS2 MD curriculum is primarily focused on the pedagogy of the diseases of the cardiovascular, renal, and respiratory systems (*R*^2^ = 0.6631). The Means-difference plot is more evenly spread out with smaller ranges of under and over predictions for all STEP1 scores.
5. Disease and Therapeutics 4 (DT4) course (Supplementary Figure 1E, Figure 4E): the final course of the MS2 MD curriculum is primarily focused on the pedagogy of the diseases of the genitourinary, gastrointestinal, and endocrine systems (*R*^2^ = 0.5917). The Means-difference plot for most students is more narrowly spread but shows over predictions for students performing poorly on STEP1.

### Correlation of Customized NBMEs in MS2 Courses With STEP1 Scores

**Figure 5:**
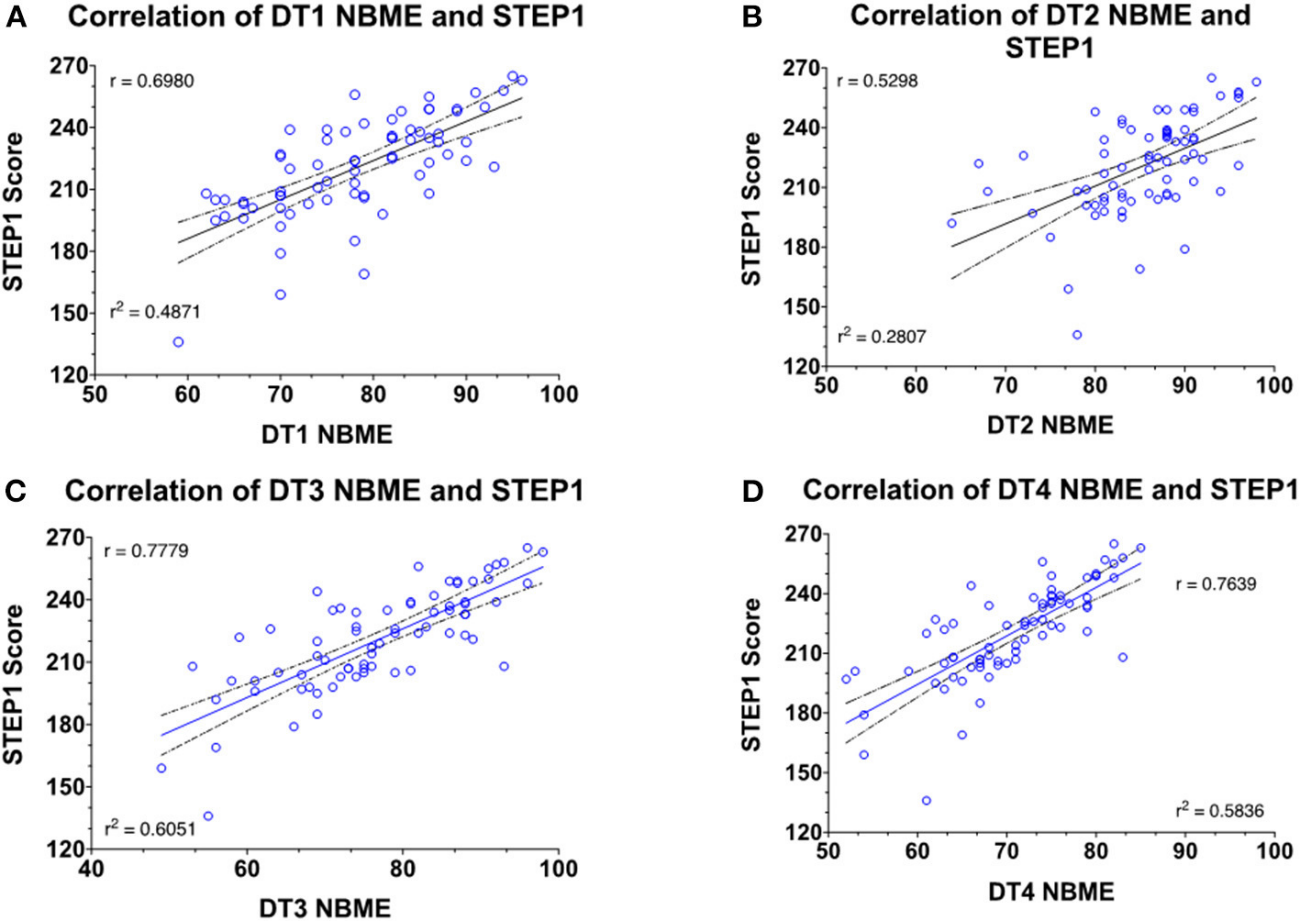
Correlation and regression analysis of the student's NBME and STEP1 scores for the Class of 2021 (*n* = 72).

For the Class of 2021, customized NBMEs were introduced as course-end comprehensive assessments for all Disease and Therapeutics courses for the MS2 curriculum. Score performance on each of those exams is correlated against students' scores, as seen in Figures 5A–D. For the class of 2020, only the DT3 course included customized NBME at the end of the course. DT3 customized NBME has the strongest linear relationship with student's STEP1 score (*R*^2^ = 0.6051).

### Prospective Analysis for the Class of 2022

**Figure 6:**
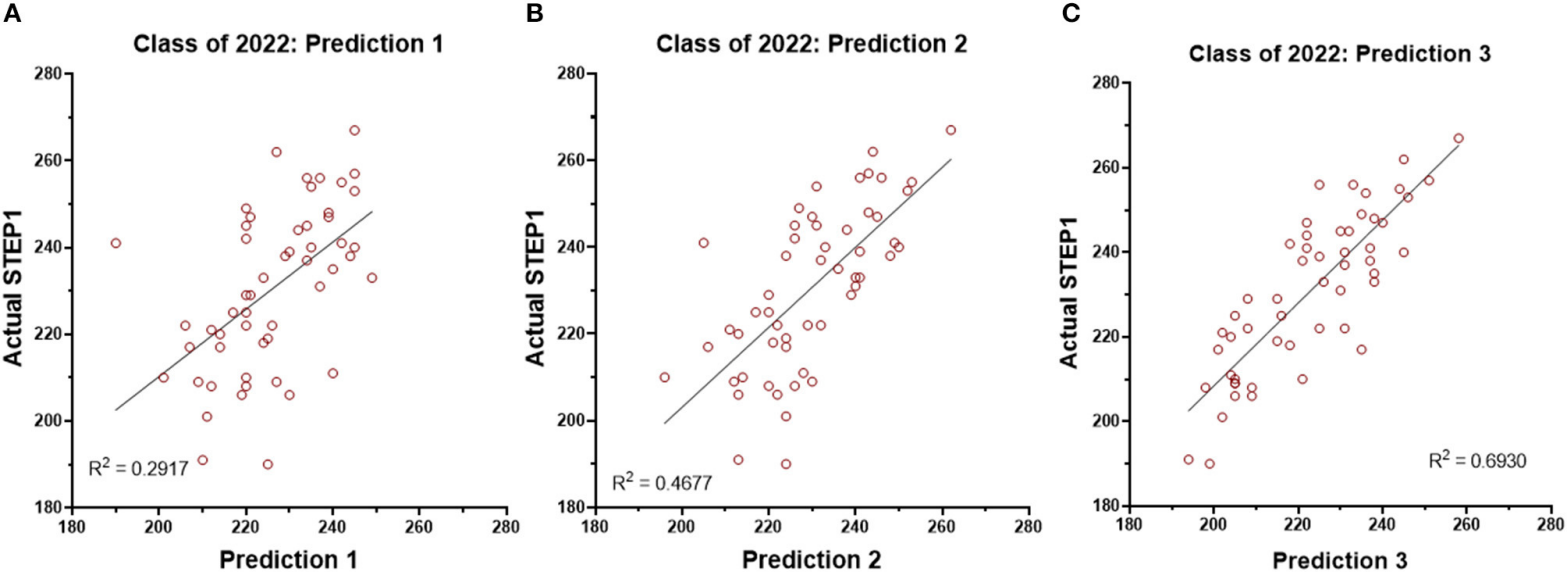
Regression analysis for the STEP1 scores and the three predictions generated for the students of the Class of 2022 (*n* = 53).

STEP1 prediction model for the Class of 2022 (*n* = 53) is based on exam and STEP1 scores of the Class of 2021. The model builds in a stepwise fashion and generates new predictions at the end of each milestone. Individual course exams are included or rejected from the prediction model based on adjusted R^2^ and the standard error of prediction. The model is used only when the adjusted R^2^ is greater than 0.5. Regression analysis for each prediction vs. student's STEP1 score is shown in Figure 6.

## Discussion

In this study, we assessed the validity of pre-matriculation data and institutional assessments in identifying future performances of medical students on the USMLE STEP1 examination. Using linear regression and Bland-Altman analysis, we were able to quantify the ability of MCAT/GPA and our pre-clerkship assessments in predicting USMLE STEP 1 scores. Our results indicate that most year one and two institutional assessments do not reliably predict students' performance on the USMLE STEP 1. Specifically, student's scores on pre-clerkship assessments lead to an overpredicted USMLE STEP 1 score. This is disadvantageous for the faculty and the administrators, who are unable to identify high-risk students and provide effective individualized interventions. Moreover, medical students engaging in self-reflection are unable to self-identify areas of needs and opportunities.

The first key finding of this study is the unreliability of MCAT and GPA in predicting USMLE STEP1 performance. When used alone, either has minimal predictive value for STEP1 performance of our students. Even combined, their correlation with STEP1 is weak at best. Similar observations were made in our earlier studies (3). However, the prospective application of prediction modeling in this study shows that traditionally used pre-matriculation data may not be generalized across institutions. Studies pooling data samples from multiple institutions or conducted at schools with higher mean MCAT scores may inflate the reliability of pre-matriculation metrics for individual institutions. In recent years, others have also questioned the value of standardized pre-matriculation assessments (5) and our results highlight the need for a comprehensive, 360° evaluation of candidates for admission into the M.D. program.

One possible reason for the lack of STEP1 predictive value of MCAT and undergraduate GPA in our study could be the evolution of these standardized tests themselves. In recent years, STEP1 has moved away from simple recall of facts to a more rounded assessment of problem-solving skills in medical knowledge, biostatistics, evidence-based medicine, and medical ethics. We should note that this data does not suggest that MCAT or GPA have no role in predicting performance on standardized tests like STEP1. These parameters appear to have predictive value for the cohort, just not for the individual. The average MCAT for students matriculating into JCESOM, Marshall University, is around the 50^th^ percentile, and so is the average for our USMLE STEP1 scores. One notable limitation of this study is only BCPM GPA is used in the predictive modeling. Due to the small sample size of students completing a post-baccalaureate masters' program in the cohort, GPA from these masters' degree programs is not included–hence, GPA used may not reflect the actual coursework success of some students.

The traditional 2 x 2 curricular model delivers information that limits the integration of foundational sciences with clinical application. Our organ-system-based MS1 curriculum is focused on the pedagogy of the normal structure and function of the human body, while the MS2 curriculum covers principles of disease and therapeutics. This design narrows the scope of assessments of each course, limiting them to only the covered disciplines. One argument in favor of this design is that students can learn each organ system twice. But, in practice, it creates informational compartments where MS1 courses cannot adequately assess complex problem-solving skills due to lack of context of the disease process. Effectively, the organ-system-based MS1 curriculum is a reorganization of the three classical MS1 disciplines, biochemistry, physiology, and anatomy. This setup renders most first-year assessments to memorization and recall of facts, ranked lower on Bloom's cognitive scale (11). This assessment style fails to utilize the high levels of Bloom's taxonomy, application, and analysis, which are the mainstays of USMLE STEP 1 questions (12).

Unsurprisingly, the second key finding of our study is that performance on most Pre-clerkship course assessments poorly correlates with students' actual USMLE STEP 1 scores. Most notably, Bland-Altman analysis reveals significant over-prediction for students underperforming on STEP1. Additionally, within the first year, significant instructor and discipline variability are readily apparent. Internal assessments with low variance led to predictions that fail to accurately identify high-risk students; therefore, students fail to receive early interventions to prevent failure on USMLE STEP 1. These shortcomings inflate the student's sense of preparedness, who often show resistance to academic support—as they are “doing well” in the curriculum.

Theoretically, predictions regarding students' performance on USMLE STEP 1 should be more accurate in the second year of pre-clerkship education because assessments should integrate basic science and clinical correlations. However, performances on internal assessments continue to over predict performance on USMLE STEP 1. This is again disadvantageous to students' ability to perform self-directed learning. Notably, both year 1 and 2 courses are remarkably similar in their design and delivery of instruction. Most of the content in these courses is delivered via lectures and assessed on a biweekly basis on course exams. To provide clinical context, case-based and large group discussions are included in courses from both years. However, there are few opportunities for students to apply their knowledge to clinical problems and receive formative feedback from assessments focused on such skills.

The practice of medicine involves life-long learning skills with the ability to integrate and apply information acquired over time and from a variety of sources. The education literature overwhelmingly shows the value of assessment in student learning (13). Formative assessment and assessment *for* learning are critical to providing learner feedback (14–16). More importantly, the quality of assessment drives the quality of learning. Increasingly, the board examinations assess the students' ability to apply foundational concepts to real-world clinical scenarios across multiple disciplines. For most pre-clerkship courses, faculty members plan assessments *after* the instruction. Most pre-clerkship assessments at the JCESOM rely on instructors submitting two or three questions/instruction hours. Regardless of pedagogy, including lecture-based, independent learnings, or case-based instructions, the assessment of the learner relies heavily on questions submitted after the fact, and commonly, these assessment items assess discipline-specific facts presented during instruction. This approach silos the complex interplay of human health and disease and conditions learners to memorize the facts as presented–impeding their ability to synthesize higher-order concepts and apply these concepts to clinical problem-solving. The learner is conditioned to seek the “buzz word” associated with a disease or a process rather than truly appreciating the complexity of the information presented.

Student performance on assessments from DT3 shows the most significant correlation with STEP 1 scores, which accounts for 66% variability in student's scores. The key differences between this second-year course and the remainder of the curricular courses include differences in pedagogy and assessment. As per the constructive alignment theory, the course emphasizes outcomes rather than content. Course objectives for the students include being able to solve clinical problems in the USMLE vignette format in both formative and summative assessments. This integrated course, with emphasis on pathophysiology and pharmacology of the cardiovascular, renal, and respiratory systems, emphasizes the application of the interplay of these disciplines. This is achieved primarily through student acquisition of foundational knowledge via lectures or independent learnings, and application of this knowledge during low-stakes team-based learning (TBL) activities. All assessment items in these TBLs are written by the instructors as a team and mimic the complexity and length of board-style questions. DT3 summative assessments overall also rank higher on the modified Blooms or the SOLO scale (17) with a greater number of critical-thinking and problem-solving questions. Notably, the Class of 2020 DT3 was the only course in the pre-clerkship M.D. curriculum to utilize a cumulative NBME assessment at the end of the course. This assessment utilizes multiple-choice questions that resemble the format of USMLE STEP 1 questions and provides performance feedback to students and faculty. Cumulative assessments may help students retain information for longer, as opposed to “binge and purge” of biweekly assessments of other courses. Additionally, cumulative assessments may aid in improved synthesis and integration of information, as these exams are likely to focus on key concepts rather than minute details. While the latter correlation allows faculty to identify high-risk students more accurately, the timing of intervention is not advantageous. Specifically, students complete this course late into the second year after USMLE STEP 1 preparation has commenced and the time to remediate areas of weaknesses is limited.

Identifying the need for institutional action and need for improved prediction of USMLE STEP1 scores, starting with the Class of 2021, and cumulative, customized NBME assessments are included in all organ-system-based courses of the MS2 curriculum. These comprehensive exams better correlate with STEP1 performance than traditional course exams (18). As before, we believe that cumulative assessments aid student's ability to synthesize and retain information for longer. Additionally, faculty can provide early interventions and focus resources on high-risk students, and medical students are able to use performance feedback to recognize areas of strengthens and weaknesses to guide their preparation for USMLE STEP 1. It is important to note, however, that not all NBMEs are similarly correlative. Our analysis indicates that this is due to differences in course design and assessments. Course(s) not dedicating enough time to integration of first- and second-year disciplines are not able to choose higher-order questions from the NBME question bank. This situation is not dissimilar to institutional assessments, i.e., customized NBME assessments may not be superior in themselves; it's the type of pedagogy, level of integration, and quality of formative assessments in the courses that appear to drive the outcome of reliable predictions of student performance.

The final key finding of this study is the validity and reliability of our STEP1 prediction model generated using targeted assessments, including the course-end NBMEs. As opposed to the retrospective model published by us (3) and others (19, 20), this model is effective prospectively. The model is deployed to identify at-risk students early and provide intervention if needed. Struggling students are identified early in the fall semester of MS2 of the M.D. curriculum and offered academic remediations, as needed. The reliability and reproducibility of our model are demonstrated with STEP1 scores for the Class of 2022, where multiple students were offered early assistance based on our prediction modeling.

In conclusion, our results show that pre-matriculation data do not significantly predict a students' USMLE STEP1 performance. Using progressive, stepwise prediction modeling, we find that most MS1 course assessments show only a weak correlation with USMLE STEP1 scores. This correlation improves for MS2 courses, but this is not uniformly applicable. The most reliable prediction model is based on assessments of only one class, i.e., three examinations. The model accuracy is also confirmed in prospective data analysis of student's taking STEP1 in the next academic year. These results go beyond performance on USMLE STEP1. They show us the insufficient reproducibility of the knowledge acquired and tested during a traditional 2x 2 M.D. curriculum. Lack of clinical context in the MS1 curriculum and failure to reconnect to foundations in the MS2 curriculum renders our assessments detail-focused, and lower on the modified Bloom's scale. Students often memorize facts in two-week bursts and often fail to understand the interplay and crosstalk of systems networks. Critical thinking and problem-solving skills are key to the successful practice of medicine. These skills require the application of foundational concepts to clinical problem-solving, which in turn are fostered by assessments integrating concepts across disciplines and systems.

## Data Availability Statement

The original contributions presented in the study are included in the article/Supplementary Material, further inquiries can be directed to the corresponding author/s.

## Ethics Statement

This study (IRB # 1630008-1) has been approved by the Marshall University Institutional Review Board under the exempt approval status.

## Author Contributions

NP: conceptualization and writing—original draft preparation. MM and BM: writing—review and editing. MM: data analysis. All authors have read and agreed to the published version of the manuscript.

## Conflict of Interest

The authors declare that the research was conducted in the absence of any commercial or financial relationships that could be construed as a potential conflict of interest.

## Publisher's Note

All claims expressed in this article are solely those of the authors and do not necessarily represent those of their affiliated organizations, or those of the publisher, the editors and the reviewers. Any product that may be evaluated in this article, or claim that may be made by its manufacturer, is not guaranteed or endorsed by the publisher.
