# Supplementary material for: Validity and Reliability of Pre-matriculation and Institutional Assessments in Predicting USMLE STEP 1 Success: Lessons From a Traditional 2 x 2 Curricular Model
Source: Front Med (Lausanne). 2022 Jan 27;8:798876. doi: 10.3389/fmed.2021.798876 (PMC8829749; doi:10.3389/fmed.2021.798876)
Supplement: Supplementary file 1 [file Data_Sheet_1.PDF]

Supplementary Materials for:

Validity and Reliability of Pre-Matriculation and Institutional Assessments in Predicting USMLE STEP 1 Success: Lessons from a Traditional 2 x 2 Curricular Model

[1]Nitin Puri, MD, PhD; [1]Michael McCarthy, [1]MS; Bobby Miller[1], MD

[1]Department of Medicine and Medical Education, Marshall University Joan C. Edwards School of Medicine, Huntington, WV, USA, 25755

Running title: Predicting STEP1 & Identifying at-risk students

Corresponding author:
Nitin Puri, MD, PhD
Associate Dean, Medical Education
Joan C Edwards School of Medicine
Marshall University
1600 Medical Center Drive, 3411
Huntington, WV, 25755
Office: 304-691-8828
Fax: 304-691-1726
Email: purin@marshall.edu

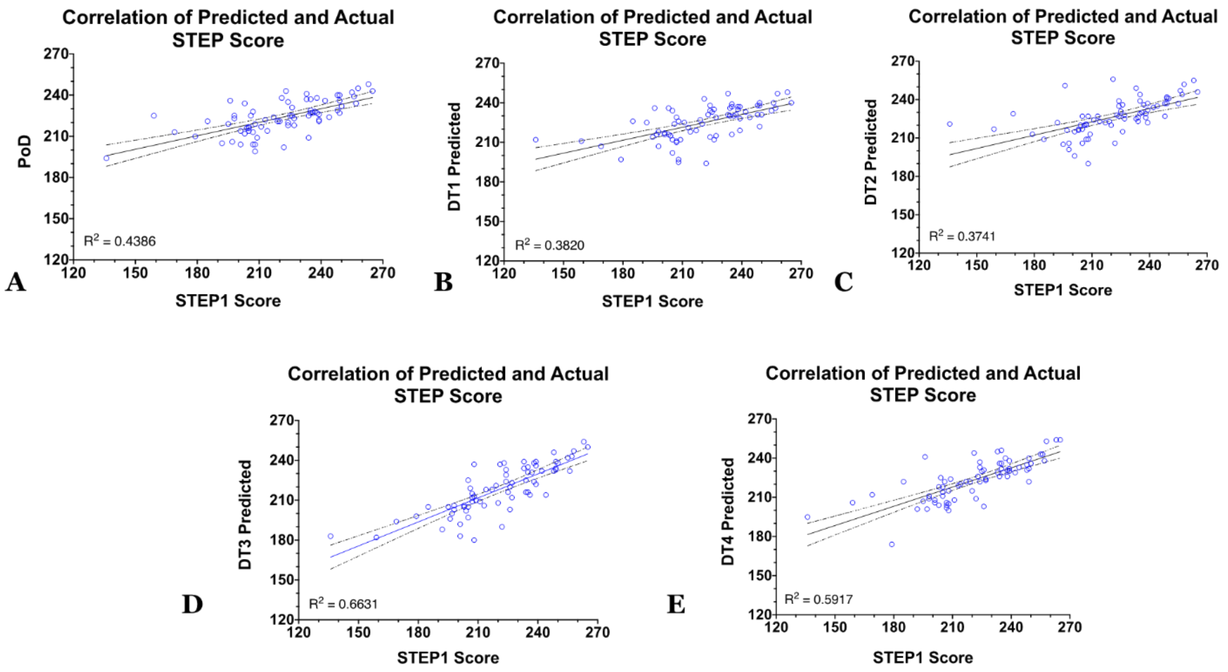

Supplementary Figure S1: Correlation and regression analysis actual vs predicted STEP1 scores at the end of each MS2 course for the Class of 2021 (n = 72).
