# Supplementary material for: Validity and Reliability of Pre-matriculation and Institutional Assessments in Predicting USMLE STEP 1 Success: Lessons From a Traditional 2 x 2 Curricular Model
Source: Front Med (Lausanne). 2022 Jan 27;8:798876. doi: 10.3389/fmed.2021.798876 (PMC8829749; doi:10.3389/fmed.2021.798876)
Supplement: Supplementary file 2 [file Data_Sheet_2.docx]

**Supplemental Methods—**

Data from the Class of 2021 is used to generate the STEP1 prediction model at each milestone (E = exam; all exams are institutionally developed unless noted otherwise) —

1. Pre-matriculation: Predicted STEP1 score (PSS) = 51.3064+(0.5342*First MCAT) +(1.2277*BCPM GPA)
2. End of course 1, MS1 (Elements of Medicine): PSS = 95.90538+(0.68099*E1) +(0.38661*E2) +(0.08675*E3) +(0.35345*E4)
3. End of course 2, MS1 (Structure and Function 1): PSS = 93.5817+(0.334*E1) +(0.8754*E2) +(0.2176*E3)
4. End of course 3, MS1 (Structure and Function 2): PSS = 72.5436+(0.884*E1) +(0.2817*E2) +(0.5207*E3)
5. End of course 4, MS1 (Structure and Function 3): PSS = 112.490246+(1.077676*E1) +(-0.007033*E2) +(0.268371*E3)
6. End of course 5, MS1 (Structure and Function 4): PSS = 123.4593+(0.6358*E1) +(0.5348*E2)
7. End of course 1, MS2 (Principles of Disease): PSS = 64.7813+(1.0024*E1) +(0.2859*E2) +(0.5547*E3)
8. End of course 2, MS2 (Disease and Therapeutics 1): PSS = 56.7937+(0.8985*E1) +(1.0028*E2)
9. End of course 3, MS2 (Disease and Therapeutics 2): PSS = 42.9588+(1.2228*E1) +(0.8839*E2)
10. End of course 4, MS2 (Disease and Therapeutics 3): PSS = 90.9247+(0.5872*E1) + (-0.009423*E2) + (1.0778*NBME score)
11. End of course 5, MS2 (Disease and Therapeutics 4): PSS = 74.2407+(0.6616*E1) +(1.0772*E2)

STEP1 prediction model for the Class of 2022 (n = 53) is based on exam and STEP1 scores of the Class of 2021. The model builds in a stepwise fashion and generates new predictions at the end of each milestone. Individual course exams are included or rejected from the prediction model based on adjusted R^2^ and the standard error of prediction. The model is used only when the adjusted R^2^ is greater than 0.5 and is presented here—

1. Predicted STEP1 score at the end of PoD—**Prediction 1**—[((SF4Exam1)*0.8884)+((POD2)*1.2103)+45.2093]; SE = 20.2518 (R^2^ = 53.48)
2. Predicted STEP1 score at the end of DT1—**Prediction 2**—[((SF4Exam1)*1.0502)+((DT1NBME)*1.2860)+30.8867]; SE = 19.9711 (R^2^ = 57.65)
3. Predicted STEP1 score at the end of DT3—**Prediction 3**—[(DT3Exam1)*0.9499+(DT3NBME)*0.9078+78.8965]; SE = 12.14 (R^2^ = 66.88)
